# An unusual presentation of developmental anomalies of the cardiovascular system including tetralogy of fallot, double outlet right ventricle, patent foramen ovale and persistent right aortic arch in a Friesian calf

**DOI:** 10.1186/s12917-020-02439-8

**Published:** 2020-06-30

**Authors:** Aine McManus, Tim Moloney, Pamela Kelly, Conor Rowan, Cliona Skelly, Catherine I. McAloon

**Affiliations:** 1 School of Veterinary Medicine, University College Dublin, Belfield, Dublin 4, Ireland; 2 Section of Pathology, School of Veterinary Medicine, Veterinary Sciences Centre, University College Dublin, Belfield, Dublin 4, Ireland; 3 Clinical Unit of Diagnostic Imaging, Vetmeduni, Vienna, Austria; 4 Section of Diagnostic Imaging, School of Veterinary Medicine, Veterinary Sciences Centre, University College Dublin, Belfield, Dublin 4, Ireland; 5 Section of Herd Health and Animal Husbandry, School of Veterinary Medicine, Veterinary Sciences Centre, University College Dublin, Belfield, Dublin 4, Ireland

**Keywords:** Heart, Congenital abnormalities, Renal agenesis, Calf, Double outlet right ventricle, Ventricular septal defect

## Abstract

**Background:**

Congenital heart diseases are occasionally encountered in the bovine species. Ventricular septal defects (VSD) and atrial septal defects (ASD) are reported to be the most common; however, a vast collection have been reported [1, 2]. Congenital heart diseases is thought to represent less than 3% of all congenital abnormalities in calves [3]. Various cardiac anomalies arise due to defective embryologic development such as defects of the septae or the cardiac chambers [2]. The exact aetiology of these congenial heart anomalies remains to be fully elucidated [4]. VSDs appear to be the most common congenital cardiac anomaly in calves. Other diseases can be subdivided into cyanotic (e.g. ASD or patent ductus arteriosus) and non-cyanotic (e.g. tetralogy of fallot or eisenmengers complex) [5, 6]. An exceptional presentation of an array of congenital anomalies was identified in a Friesian heifer calf. To the authors’ knowledge this concurrent collection of congenital abnormalities has never been reported in this species.

**Case presentation:**

A 3-day old Friesian heifer presented with a history since birth of regurgitation post feeding. The main finding on clinical examination was tachypnoea with a holosystolic murmur. Echocardiography identified a VSD, patent foramen ovale (PFO) (both with left to right blood flow) and tricuspid insufficiency. The calf was subsequently euthanised and underwent gross post-mortem examination. A persistent right aortic arch (PRAA) was identified. The cardiac anomalies identified on the echocardiogram were confirmed along with additional abnormalities; double outlet right ventricle (DORV), partial transposition of the great vessels, pulmonic stenosis, hypoplasia of the right branch of the pulmonary artery and right ventricular hypertrophy. The final diagnosis was Tetralogy of Fallot with DORV, PFO and PRAA. The lungs appeared oedematous and congested due to cardiac malfunction and cranioventral aspiration pneumonia. Free serous fluid was identified in the thoracic cavity. Unilateral renal agenesis of the left kidney was an incidental finding but is of note due to its coexistence with the cardiac abnormalities.

**Conclusions:**

This is an unusual case as it features numerous congenital abnormalities that appeared to negate each other allowing capability with life. To the authors’ knowledge, this collection of concurrent cardiac anomalies has not been previously reported in bovines.

## Background

Congenital heart diseases are occasionally encountered in the bovine species. Ventricular septal defects (VSD) and atrial septal defects (ASD) are reported to be the most common; however, a vast collection have been reported [1, 2]. Congenital heart disease is thought to represent less than 3% of all congenital abnormalities in calves [3]. Various cardiac anomalies arise due to defective embryologic development such as defects of the septae or cardiac chambers [2]. The aetiology of these congenial heart anomalies is yet to be fully elucidated [4]. VSD is the most commonly reported congenital cardiac anomaly in calves. Other diseases can be divided into cyanotic (e.g. ASDs or patent ductus arteriosus) and non-cyanotic (e.g. tetralogy of fallot or eisenmenger’s complex) [5, 6]. An exceptional presentation of an array of congenital anomalies was identified in a Friesian heifer calf. To the authors’ knowledge this concurrent collection of congenital abnormalities has not been previously reported in this species.

## Case presentation

A 3-day old Friesian heifer calf presented to the University Veterinary Hospital, University College Dublin, with a history since birth of milk regurgitation. The calf was noted by the owner to have a suckle reflex. Milk was observed discharging from its nostrils after feeding. The owner also noted the calf was smaller than its peers weighing only 37 kg. On clinical exam the calf was tachycardic with a heart rate of 160 beats per minute. A holosystolic murmur was auscultated with the point of maximal intensity at the left heart base (grade 6/6). A pre-cordial thrill was detected. The jugular veins appeared normal. Mucous membranes were pale pink, with no evidence of cyanosis. Dehydration status was less than 5%. The calf was tachypnoeic; respiratory rate was 60 breaths per minute with increased lung sounds auscultated throughout the lung field. No wheezes or crackles were audible. Body temperature was within normal range (38.8 °C). The calf was bright, alert and responsive with normal mentation and locomotor system.

**Fig. 1:**
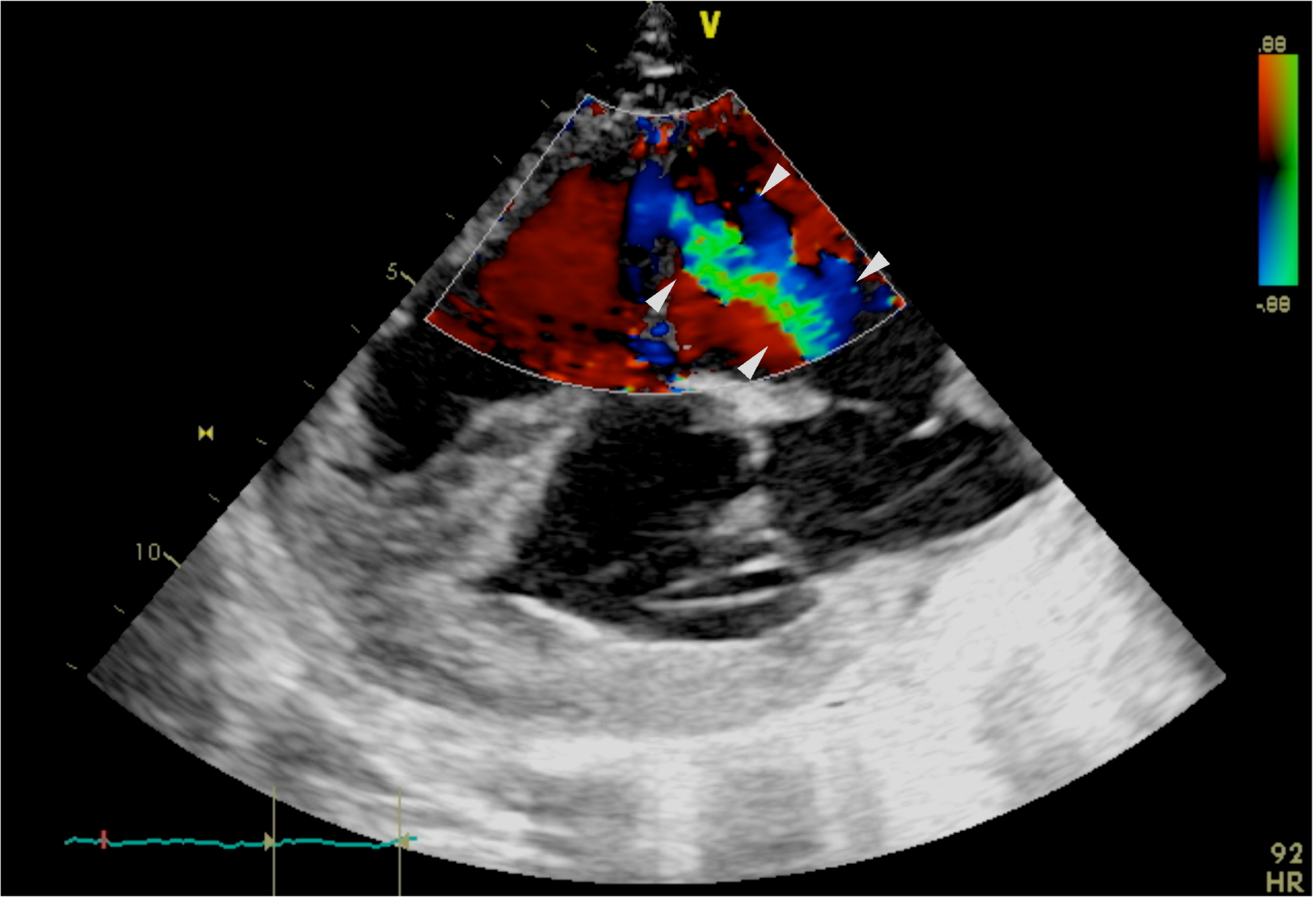
Caudal long cardiac axis (right side) 4 chamber view with spectral Doppler identifying the high velocity turbulent tricuspid insufficiency (demarcated by white arrowheads). Images obtained at the 4th intercostal space, due to abnormalities angulation was non-standardised and optimised reactively to images displayed; due to abnormalities angulation was non-standardised and optimised reactively to images displayed

**Fig. 2:**
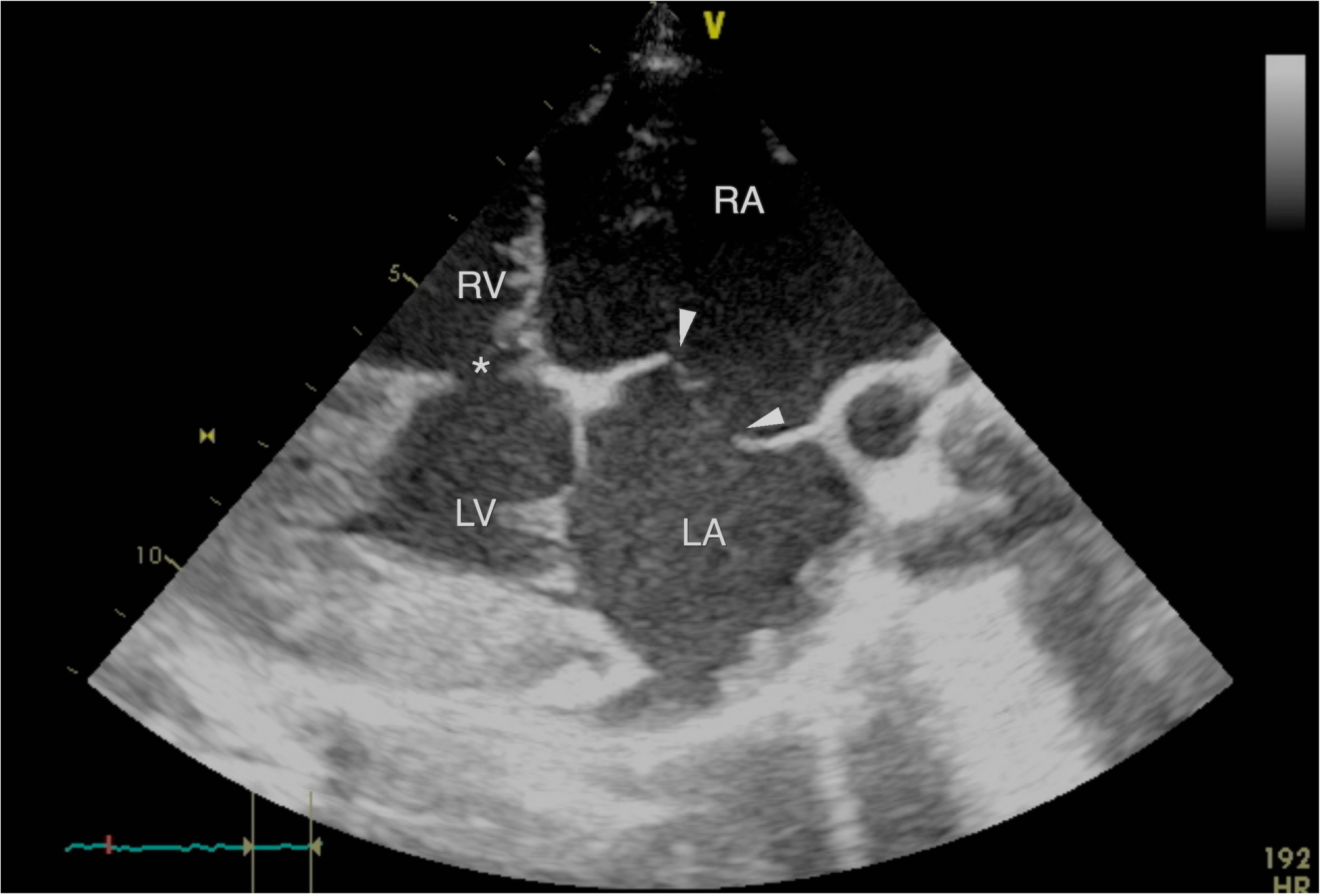
Caudal long cardiac axis (right side) 4 chamber view displaying the patent foramen ovale (white arrow heads) resulting in communication between the distended right atrium (RA) and left atrium (LA); and the ventricular-septal defect (*) resulting in communication between the distended right ventricle (RV) and left ventricle (LV); due to abnormalities angulation was non-standardised and optimised reactively to images displayed. Images obtained at the 4th intercostal space, due to abnormalities angulation was non-standardised and optimised reactively to images displayed; due to abnormalities angulation was non-standardised and optimised reactively to images displayed

An echocardiogram was performed (machine Acuson Sequoia, probe 3V2C) the following day to investigate the audible heart murmur. The calf was tachypnoeic and restless throughout the unsedated exam. Echocardiography was performed at the right and left cranial thorax (4th to 6th intercostal space). Angulation and position of the probe was variable and optimised reactively to the images displayed on the screen. Adherence to a previously reported protocol resulted in non-diagnostic images [6]. The right atrium and right ventricle were severely enlarged. Both atrial and ventricular septa were displaced to the left. The right atrium measured approximately twice the maximal transverse diameter of the left (5.4 cm compared with 2.6 cm). Subjective high velocity turbulent tricuspid regurgitation was identified and there was incomplete coaptation of the tricuspid leaflets (Fig. 1). There was a thin communication in the proximal interventricular septum, with left to right flow identified (velocity not recorded). Flow from the left atrium to the right atrium via the foramen ovale was identified (peak velocity measured 3.6 m/s). Definitive accurate recording of spectral Doppler values for all flow was not feasible due to the interference both of calf movement and tachypnoea, and the suboptimal angle of insonation. Given the severe nature of the changes, the poor prognosis and the tachypnoeic nature of the calf; aggressive physical restraint nor chemical restraint was determined to be counterproductive and the echocardiogram was concluded. The conclusive echocardiographic findings were: VSD, patent foramen ovale (PFO) (both with left to right blood flow) and tricuspid insufficiency (Fig. 2).

Given the severity of the cardiac abnormalities, the calf was subsequently euthanised via intravenous injection of pentobarbital sodium (Release, 5 ml/10KG BW).

**Fig. 3:**
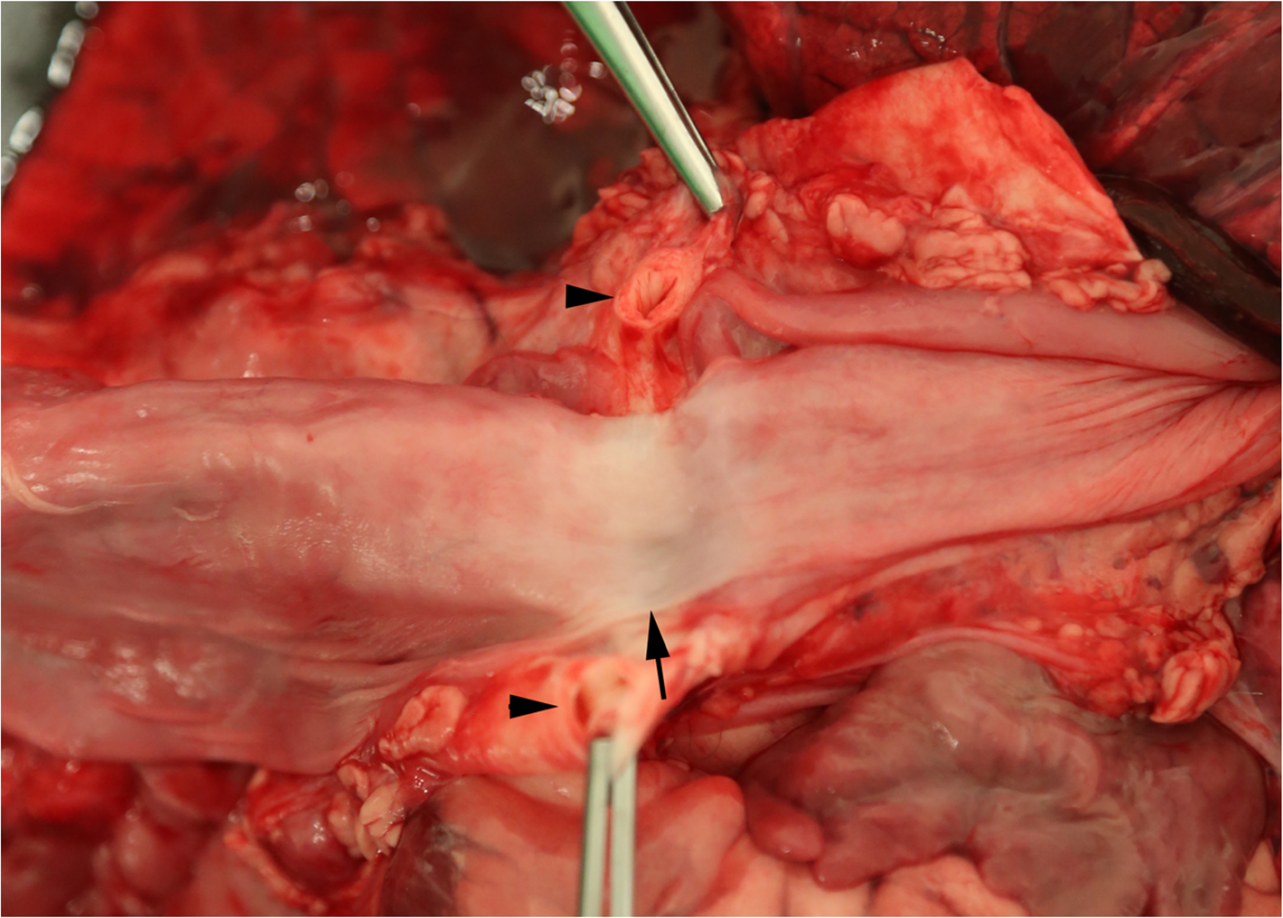
Photograph of the oesophagus and surrounding muscle and connective tissue, showing the ductus arteriosus (arrow heads) and the annular stricture of the oesophagus (arrow)

**Fig. 4:**
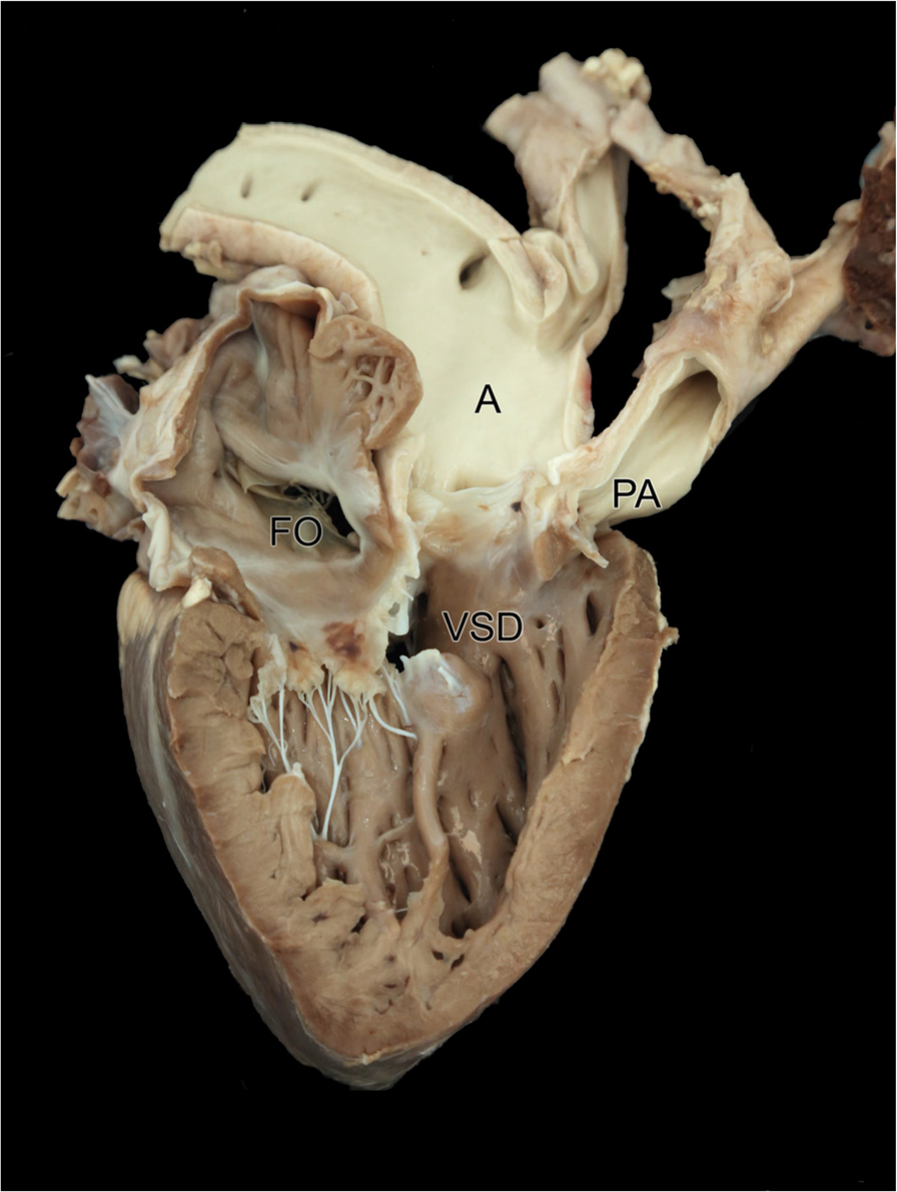
Photograph of the right atrium and right ventricle showing the persistent foramen ovale (FO), large subaortic ventricular septal defect (VSD), the transposed aorta (A) and the hypoplastic pulmonary artery (PA). Both the aorta (A) and pulmonary artery (PA) are exiting from the right ventricle (double outlet right ventricle)

Post-mortem examination revealed further congenital abnormalities not identified on echocardiogram and clinical exam. The aorta emerged from the right ventricle together with the pulmonary artery, (double outlet right ventricle) (Fig. 4) and continued on the right side of the oesophagus (persistent right aortic arch). There was entrapment of the oesophagus by the ductus arteriosus (ligamentum arteriosum), compressing it against the trachea (Fig. 3). The oesophagus proximal to this was moderately dilated. The pulmonic valve was stenotic and the pulmonary artery hypoplastic. There was mild dilation of the hypoplastic pulmonary artery just proximal to the pulmonic valve and distal to the division of right and left branch of the pulmonary artery. The right branch of the pulmonary artery was markedly hypoplastic compared to the left branch.

The foramen ovale was large and patent, measuring a maximum diameter of 30 mm (Fig. 4). A large 20 mm diameter VSD was present ventral to the aortic and tricuspid valves. The tricuspid valve was of normal appearance. There was hypertrophy of the right ventricular wall. The left ventricular free wall measured 15 mm and the right ventricular free wall measured 10 mm (3:2). (Fig. 4). The left atrial diameter was grossly normal.

The thoracic cavity contained approximately 100mls of serosanguinous fluid. There was a mild increase in pericardial fluid. The pericardial sac was of normal thickness, the lungs were diffusely heavy and wet (oedema). The cranial lung lobes were dark red, collapsed and sank in formalin. There was agenesis of the left kidney. The right kidney was grossly normal.

Histological examination of lung tissue revealed marked pulmonary oedema, increased alveolar macrophages and dilated lymphatics. The cranial lung tissue revealed chronic active neutrophilic and histiocytic bronchopneumonia (aspiration). The right kidney was examined histologically and was normal.

Given the findings of pulmonic stenosis, a VSD, right ventricular wall hypertrophy, an overriding aorta which exited from the right ventricle together with the pulmonary artery, a PFO and entrapment of the oesophagus by the ductus arteriosus a final diagnosis of Tetralogy of Fallot with DORV, PFO and PRRA was given.

In this case report we present an unusual array of congenital heart defects in a calf. Although described individually in the literature and in various combinations, this combination of abnormalities is novel.

A persistent right aortic arch (PRAA) was identified at post-mortem examination that was responsible for the main presenting clinical sign of milk regurgitation. Major development changes occur in the aortic arch arteries in early gestation, with the establishment of separate venous and arterial circulations [7]. In the normal embryo, the left 4th aortic arch artery forms part of the arch of the aorta and the right 4th aortic arch artery forms the proximal segment of the right subclavian artery. However, in the PRAA anomaly, the aortic arch forms from the right 4th aortic arch artery instead of the left, resulting in formation of the aorta to the right rather than the left of the oesophagus. A vascular ring is formed by the right aortic arch, ductus arteriosus (later ligamentum arteriosum) and the base of the heart ventrally. The vascular malposition leads to encircling and compression of the oesophagus with resultant clinical signs [7]. The PRAA is recognised as a common vascular ring anomaly in dogs [8] and has been previously reported in bovines [9].

A large subaortic, perimembranous VSD was also identified on echocardiography and confirmed at post-mortem examination. This VSD was located just below the aortic valve and extended to involve both the membranous and muscular septum. This anomaly occurs due to a failure of complete development of the ventricular septum, most commonly the membranous part of the inter-ventricular septum. The exact position and size of the septal defect can vary. A high and small septal defect is more common [3]. VSDs result in a left to right shunt through the opening and resultant hypertrophy of the ventricles. Ventricular septal defect (VSD) is the most common cardiovascular abnormality in bovine neonates and often occurs with other congenital abnormalities as is the case in this calf [2].

Double outlet right ventricle (DORV) was also identified at post-mortem examination. This is a rare conotruncal malformation where both the aorta and the pulmonary artery arise from the right ventricle [10]. Cases of DORV are categorised according to the presence or absence of pulmonic stenosis and the location of the VSD (subaortic, subpulmonary, doubly committed, noncommitted). Prosek et al. (2005) discussed how DORV with a subaortic VSD, pulmonary stenosis, and greater than 50% aortic override resembles both morphologically and physiologically a tetralogy of Fallot [11]. DORV with a subpulmonic VSD and no pulmonary stenosis (Taussig-Bing anomaly) resembles complete transposition of the great arteries. In this calf, there was pulmonary stenosis (valvular and branch peripheral pulmonic stenosis) and a subaortic VSD. Pulmonary stenosis is reported in 18–68% of humans with a subaortic VSD and DORV [12].

Right ventricular wall hypertrophy was also identified at post-mortem examination. Right ventricular wall hypertrophy is reported in cases of pulmonic valve stenosis where increased effort is required to pump blood through the narrowed pulmonic valve as in this case.

Echocardiography of the heart identified a PFO which was confirmed by post-mortem examination. During foetal development, the common atrium is partitioned by the septum primum and the septum secundum membranes with a foramen existing within the wall; the foramen ovale. A portion of the septum primum forms a valve like structure for the foramen ovale [7]. This anatomical feature of the foetal circulation allows oxygenated blood to pass directly from the right atrium to the left atrium (and bypass the non-functional lungs). The valve of the foramen ovale is kept open by a higher pressure of blood in the right atrium than the left atrium. At birth, pressure changes (reduced right atrial pressure) result in the septum primum pressing against the septum secundum closing the foramen ovale. Functional closure should occur quickly whereas anatomical closure occurs gradually over the first year of life. As in this calf the presence of a PFO meant functional closure did not occur. This may be due to; failure of the septum to close due to pressure abnormalities or defective development of either the foramen primum or secundum [3].

Differentials for tricuspid insufficiency include: myxomatous atrioventricular valvular degeneration (endocardiosis), bacterial endocarditis; dilated right ventricle with secondary dilation of the annular ring; secondary to pulmonic stenosis and tricuspid valve dysplasia [13]. Given the young age and relatively well-defined leaflets; endocarditis and endocardiosis were considered unlikely at the time of echocardiography. Retrospectively given the normal appearance of the tricuspid valve on post-mortem; annular dilation secondary to right ventricular enlargement most likely accounts for the severe insufficiency in this calf. Whether this right ventricular dilation was secondary to the VSD or the pulmonary stenosis or a combination of both conditions remains speculative.

The lack of clinical cyanosis was unusual. This was likely due to oxygenating blood exiting from the left ventricle via the VSD to reach the transposed aorta, enabling adequate oxygenated blood to reach the body. Regardless, long term survival in this case was unlikely; this calf already had aspiration pneumonia secondary to the PRAA and had evidence of right ventricular hypertrophy. We speculate that further hypertrophy of the right ventricle in the future would reverse the blood flow across the VSD (Eisenmenger’s syndrome) resulting in left ventricular dilation and severe hypoxemia.

We speculate that the right sided cardiac modelling and DORV resulted in an altered cardiac position that accounted for the challenging nature of the echocardiography and the need for non-standardised angulation/rotation of the probe to acquire diagnostic images.

The pulmonary oedema noted both grossly and histologically is considered to be of cardiogenic origin given the malformation of the heart. However, with pulmonary oedema the most common gross lesion seen is dilation of the left atrium, this was not noted in this case. The lack of left atrial dilation is attributed to the age of the calf (3 days); if this calf lived for a longer period of time the left atrium would most likely become dilated.

A further congenital abnormality identified was agenesis of the left kidney. The right kidney was of normal size with normal histological features suggesting normal functionality. Renal agenesis is the absence of one or both kidneys. It is always accompanied by absence of the ipsilateral ureter. Aplasia of the ipsilateral reproductive tissues may accompany the renal agenesis. Renal agenesis is usually an incidental finding as long as the other kidney is present and functioning well, however in some cases of unilateral renal agenesis the contralateral kidney is hypertrophied to compensate. Bilateral renal agenesis is inconsistent with postnatal life. There is a familial disposition for unilateral renal agenesis in certain breeds of dog [14] and it is the most common congenital kidney defect of pigs [14], but it is rarer in cattle [15].

## Conclusion

This complex arrangement of congenital abnormalities may have remained undetected had it not been for the unusual presenting sign of milk regurgitation. The calf was not cyanotic at presentation; the combination of anomalies enabled the heart to deliver oxygenated blood around the body. It is unusual that the combination of cardiac abnormalities encountered almost negated each other allowing compatibility with life. It is likely that impending death was inevitable due to the massive stress on the heart and resulting hypertrophy, in combination with the aspiration pneumonia.

This report highlights an unusual collection of abnormalities in an apparently functional calf. To the authors’ knowledge, this collection of concurrent cardiac anomalies has not been previously reported in bovines. This case report will add to the clinical, diagnostic and pathological findings in calves with congenital cardiac disease.

## Data Availability

All material relevant to the case report is included as images and freely available.

## Abbreviations

VSD: Ventricular septal defects
ASD: Atrial septal defects
PRAA: Persistent right aortic arch
PFO: Patent foramen ovale
DORV: Double outlet right ventricle
